# Investigating the Accuracy of the Digihaler, a New Electronic Multidose Dry-Powder Inhaler, in Measuring Inhalation Parameters

**DOI:** 10.1089/jamp.2021.0031

**Published:** 2022-06-10

**Authors:** Henry Chrystyn, Dinesh Saralaya, Anil Shenoy, Sophie Toor, Kari Kastango, Enric Calderon, Thomas Li, Guilherme Safioti

**Affiliations:** ^1^ Inhalation Consultancy Ltd., Leeds, United Kingdom.; ^2^ Bradford Institute for Health Research, Bradford Teaching Hospitals NHS Foundation Trust, Bradford, United Kingdom.; ^3^ Respiratory Matters, Huddersfield, United Kingdom.; ^4^ Department of Statistical Operations, Everest Clinical Research, Little Falls, New Jersey, USA.; ^5^ Department of Combination Products and Devices R&D, Teva Pharmaceutical Industries, Waterford, Ireland.; ^6^ Department of Statistics, Teva Branded Pharmaceutical Products R&D Inc., West Chester, Pennsylvania, USA.; ^7^ Department of Connected Respiratory, Teva Pharmaceutical Industries Ltd., Amsterdam, The Netherlands.

**Keywords:** digital technology, dry powder inhalers, inhalation devices, lung diseases

## Abstract

**Background:**

The Digihaler^®^ is a Food and Drug Administration-approved, digital multidose dry powder inhaler with an integrated electronic module that provides patients and health care professionals with feedback on inhalation parameters, including usage, adherence, and technique. This study compared inhalation parameters measured using the Digihaler with readings made simultaneously using an inhalation profile recorder (IPR).

**Methods:**

This single-visit, open-label study enrolled children (4–17 years) and adults (18–55 years) with asthma, and adults (≥55 years) with chronic obstructive pulmonary disease (COPD). Participants made three separate inhalations using an empty Digihaler device, each measured simultaneously by the Digihaler and IPR. Inhalation profiles were downloaded from the devices at the end of the study. Inhalation parameters measured included peak inspiratory flow (PIF) and inhaled volume (inhV). The profile with the highest PIF and corresponding IPR profile were analyzed.

**Results:**

Overall, 150 participants were enrolled; inhalation data were available for 148 (50 children and 49 adults with asthma, and 49 with COPD). Mean (standard deviation [SD]) age was 39.1 (24.5) years; 51% of participants were male. Overall mean (SD) PIFs as measured by the Digihaler and IPR were 70.62 (17.73) L/min and 72.55 (19.42) L/min, respectively, with a mean percentage difference of −1.75% (95% confidence interval [CI]: −3.64 to 0.15). Mean percentage differences between the Digihaler and IPR measurements of PIF ranged from −2.97% among adults with COPD to 0.16% among children with asthma. Overall mean (SD) inhV for the Digihaler and IPR were 1.57 (0.69) L and 1.67 (0.73) L, respectively, with a mean percentage difference of −6.11 (95% CI: −8.08 to −4.13). There was a strong correlation between PIF and inhV measurements taken by the Digihaler and those taken by the IPR (Spearman's correlation coefficient = 0.96).

**Conclusions:**

Our findings confirm the ability of the Digihaler to provide accurate measurement of inhalation parameters when used by patients.

## Introduction

Inhaled therapies are the mainstay of treatment for both asthma and chronic obstructive pulmonary disease (COPD).^(1,2)^ Global recommendations emphasize the importance of medication adherence and correct inhalation technique in managing symptoms; however, both are well-recognized challenges in clinical practice.^(1,2)^

Estimates of medication adherence in asthma and COPD have been shown to vary widely.^(1–7)^ A threshold of 80% is commonly used to determine adherence.^(8–10)^ Indeed, medication adherence of >80% is associated with a lower risk of mortality and hospitalization in patients with COPD,^(11,12)^ and a higher forced expiratory volume in 1 second (FEV_1_) and lower sputum eosinophil levels in patients with asthma.^(13)^ Despite the importance of medication adherence, standard techniques such as medication reminders, adherence tracking, and patient education have proved difficult in improving long-term medication adherence, and so alternative approaches are required.^(14,15)^

Critical inhalation technique errors when using metered dose inhalers (MDIs) and dry powder inhalers (DPIs) are common.^(16,17)^ Correct DPI use is dependent upon inspiratory effort to ensure delivery of the required drug dose^(18–21)^ and not using a fast-enough inhalation has been found to contribute to poor asthma control.^(17)^ Inspiratory effort when using a DPI is difficult to monitor on an ongoing basis, yet this information would be useful in assessing inhaler technique.^(22)^

Add-on electronic adherence monitors, which can be clipped on to existing inhalers to provide a timestamp for each inhaler dose, have been available for over 25 years.^(23)^ Newer digital inhaler systems have the potential to provide automatic and continuous monitoring of dose administration and inhalation technique, with no additional burden to patients or interference with their normal inhaler use.^(15,24,25)^ Data from these rechargeable devices can be uploaded electronically through physical connections, wireless internet, or Bluetooth^®^ to form a record of a patient's ongoing inhaler use and performance.^(26)^ This information may enable personalized interventions and instant feedback to patients^(15)^; however, while many newer devices can monitor actuation and provide medication reminders, most do not assess inhalation technique.^(24)^

**FIG. 1.**
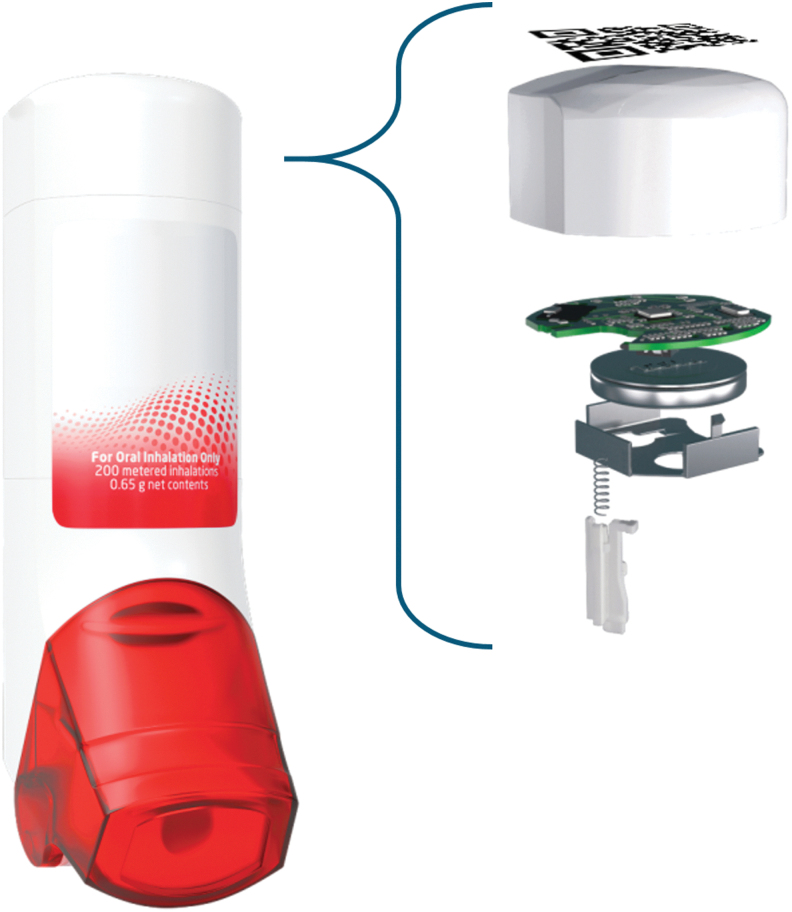
Exploded schematic view of the Digihaler.

The Digihaler^®^ is an electronic multidose DPI with fully integrated digital sensors (Fig. 1). It has received Food and Drug Administration approval as ProAir^®^ Digihaler (albuterol sulfate), ArmonAir^®^ Digihaler (fluticasone propionate), and AirDuo^®^ Digihaler (salmeterol, fluticasone propionate).^(27)^ An electronic module (eModule) is integrated within the device with sensors that record real-life use through timestamped inhalation data, including parameters that are relevant for assessment of inhalation technique.^(28)^ A microelectromechanical system sensor detects pressure changes at the top of the inhaler generated by inhalation airflow at the mouthpiece. These changes are used by the eModule to calculate inhalation parameters, including flow rate. The integrated sensors and associated electronics are in the top of the upper-case assembly of the inhaler device and, therefore, do not interfere with either the air flow or drug path within the device.

*In vitro* assessment of the Digihaler has shown that peak inspiratory flow (PIF) is the most relevant inhalation parameter in determining albuterol dose delivery,^(29)^ and a study of patients with COPD demonstrated that PIF greatly influenced the emitted dose.^(18)^ The ability to measure PIF using the Digihaler is therefore an important development in confirming that the patient made an inhalation and assessing inhalation technique.^(15)^

Before data from such devices can have clinical utility and be used for real-world assessment, it is essential to determine that the data captured are consistent with the research-based, validated external methods that identify the pressure changes inside the mouthpiece, which relate to deaggregation of the metered dose during an inhalation.^(30)^ These traditional methods to measure inhalation flow cannot be made during real-life use. The aim of this study was to identify if the eModule measurements from the Digihaler when patients make inhalations are as accurate as the traditional research-based methods. To do this, inhalation parameters measured using an empty Digihaler were compared with readings made simultaneously using an inhalation profile recorder (IPR), in children and adults with asthma, and adults with COPD.

## Materials and Methods

### Participants

Eligible participants included children (4–17 years) and adults with asthma (18–55 years; FEV_1_ <80% predicted), and adults with COPD (≥55 years; FEV_1_ <50% predicted). Participants were required to have ≥6 months history of asthma or COPD and ≥3 months experience of inhaler use before screening. Exclusion criteria included: another confounding, underlying lung disorder; a clinically meaningful comorbidity that would interfere with the study schedule or procedures, or compromise participant safety; and inability or refusal to make an inhalation using a DPI.

### Study design

This single-visit, open-label, non-interventional clinical device study was performed in two centers in the United Kingdom from December 20, 2016 to May 28, 2017. The study comprised three periods: screening, an inhalation testing date, and a follow-up phone call the following day (study day 2). Participants were either invited to take part in the study during routine standard-of-care clinic appointments or were identified using a research hospital database and invited through letter. Participants attended the investigational center; those who met the study inclusion criteria proceeded to inhalation testing during the same visit or within the next 7 days. All Digihaler devices used were empty and no pharmaceutical product or excipient was administered during the study. The study was conducted in accordance with the principles of the Declaration of Helsinki and conformed to appropriate ethical guidelines. All participants provided written informed consent. U.K. Health Research Authority ethics approval was obtained (16/YH/0346).

### Study outcomes

The primary objective was to determine the accuracy of the Digihaler by comparing PIF measurements taken using an empty Digihaler with those from an IPR. Secondary objectives included comparing inhaled volume (inhV), time to PIF (Tp), and inhalation time (Ti) between the Digihaler and IPR. Details on safety assessments can be found in Section 1.1 of the Supplementary Data.

### Study assessments

#### Baseline characteristics

Demographic data, vital signs, and concomitant medications were recorded during the inhalation testing visit. Spirometry tests were conducted in all participants except children <12 years of age. Participants with COPD completed the Medical Research Council Breathlessness Scale.^(31)^ Asthma control was assessed according to the Global Initiative for Asthma recommendations available at the time of the study.^(32)^

#### Inhalation parameters

**FIG. 2.**
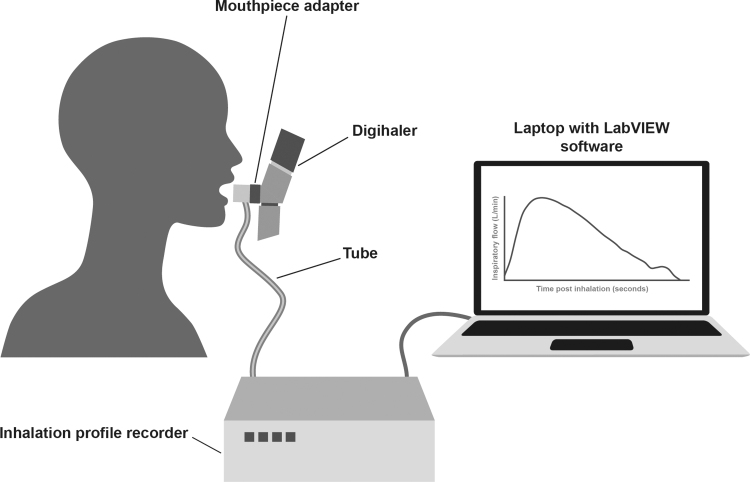
Schematic of the Digihaler inhalation profile recorder setup.

A single-use adapter (provided clean and individually wrapped) was attached to the mouthpiece of a new empty Digihaler and connected to an IPR. An IPR probe was connected to PR3202 low differential pressure sensors (Applied Measurements Ltd., Reading, UK). Data were downloaded directly from the devices and converted to inhalation flows according to the methods described previously (Fig. 2).^(33)^ Details about the IPRs have been previously described in Azouz et al.^(33)^ and are briefly detailed in Section 1.3 of the Supplementary Data. Two separate IPRs (IPR2 and IPR3) were used in this study. *In vitro* assessments were performed to verify the accuracy of these in measuring PIF. Inhalation data from the eModule of each Digihaler were downloaded after the study by appropriately designated and trained personnel using extraction software.

**FIG. 3.**
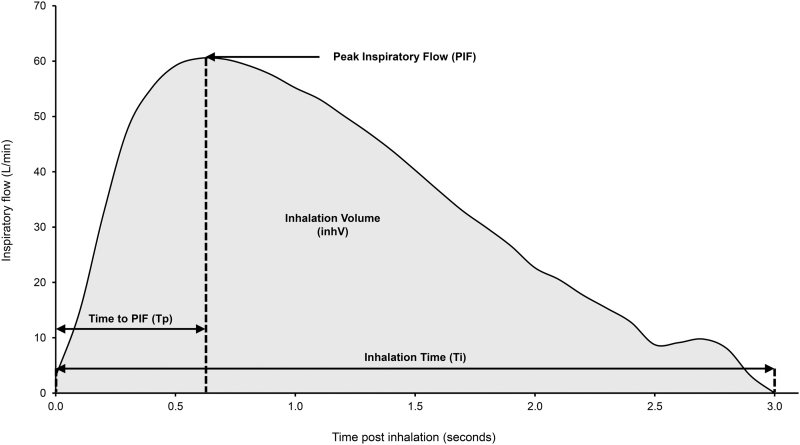
Inhalation profile showing inhalation parameters. Figure shows the inhalation profile of a 76-year-old male with chronic obstructive pulmonary disease, 1.62 m tall and weighing 56 kg. PIF = 60.5 L/min; inhV = 1.65 L; Tp = 0.6 seconds; Ti = 3 L. inhV, inhalation volume; PIF, peak inspiratory flow; Ti, inhalation time; Tp, time to PIF.

Participants were verbally trained in correct inhalation technique (Section 1.2 of the Supplementary Data) and then required to make three separate successful inhalations with a 2-minute rest period between each inhalation. The eModule of the Digihaler turns off 1 minute after opening the cap so any inhalation performed outside this time frame was discarded and the inhalation was repeated. Following each triplicate of maneuvers, the profile with the highest PIF as measured by the Digihaler was selected for analysis. Data collected from the Digihaler and IPR were regarded as paired measurements. Participant inhalation profiles were used to determine inhalation parameters, as shown in Figure 3.

### Statistical and analytical methods

No formal statistical sample size/power calculations were performed. A previous study revealed that 50 children with asthma and 100 adults (50 with asthma and 50 with COPD) were adequate to provide a reliable assessment of inhalation parameters.^(33)^ No formal hypotheses were tested during the study. Descriptive summary statistics were used. Estimation included the point estimate together with two-sided 95% confidence interval (CI), as appropriate. No adjustments for multiplicity were applied to any of the endpoints.

Participants were categorized based on respiratory condition and age (children with asthma, adults with asthma, and adults with COPD) and PIF measurements (<30 L/min, 30 to <45 L/min, 45 to <60 L/min, and ≥60 L/min). The intention-to-treat (ITT) analysis set included all enrolled participants and was used for all study population summaries.

Paired Digihaler and IPR-measured inhalation data collected from each group were compared using the Bland–Altman method^(34–36)^ and Spearman's correlation coefficient. Percentage differences between the Digihaler and IPR measurements within categories were calculated as below:

$$\begin{array}{cc} & \%difference=\\ & \frac{Digihalermeasurement-IPRmeasurement}{IPRmeasurement}x100\% \end{array}$$

A *post hoc* analysis was performed to determine the 95% credible intervals of the Bayesian posterior percentage differences between Digihaler and IPR-measured PIF.

## Results

### Participants

Overall, 150 participants were enrolled (50 each of children with asthma, adults with asthma, and adults with COPD). Age brackets of the children were 4–6 years (*n* = 10), 7–11 years (*n* = 23), and 12–17 years (*n* = 17). Four children with asthma were lost to follow-up through phone call a day after measurement of their inhalation profiles. One adult with COPD was 49 years old and did not meet the age criterion; this participant was considered a protocol violation but was included in the ITT analysis set.

Baseline characteristics are shown in Table 1. The most commonly reported comorbidities were: eczema (*n* = 24 [48%]) and food allergies (*n* = 9 [18%]) in children with asthma; depression (*n* = 17 [34%]) and allergies (*n* = 15 [30%]) in adults with asthma; and depression and hypertension (*n* = 19 [38%] each) in adults with COPD.

**Table 1.** Baseline Characteristics

Baseline characteristic	Children with asthma (*n* = 50)	Adults with asthma (*n* = 50)	Adults with COPD (*n* = 50)	Total (*N* = 150)
Mean (SD) age, years	9.8 (3.4)	40.8 (10.5)	66.8 (6.8)	39.1 (24.5)
Female, *n* (%)	20 (40)	28 (56)	25 (50)	73 (49)
Mean (SD) weight, kg	40.5 (18.3)	83.4 (22.5)	75.3 (18.1)	66.4 (27.1)
Race, *n* (%)				
White	8 (16)	27 (54)	50 (100)	85 (57)
Black or African American	1 (2)	0	0	1 (<1)
Asian	40 (80)	22 (44)	0	62 (41)
FVC, L^a^				
*N*	17	50	50	117
Mean (SD)	2.91 (0.45)	2.82 (0.87)	1.95 (0.66)	2.46 (0.85)
Median	2.86	2.78	1.86	2.42
Minimum–maximum	2.19–3.73	0.90–4.73	0.68–3.89	0.68–4.73
Percent predicted FEV_1_, %^a^				
*N*	17	50	50	117
Mean (SD)	92.5 (20.3)	61.1 (14.1)	35.7 (9.7)	54.8 (23.7)
Median	92.0	64.5	35.0	49.0
Minimum–maximum	52–133	32–79	17–49	17–133
FEV_1_, L^a^				
*N*	17	50	50	117
Mean (SD)	2.43 (0.41)	1.89 (0.62)	0.89 (0.30)	1.54 (0.76)
Median	2.55	1.77	0.90	1.40
Minimum–maximum	1.87–3.27	0.63–3.18	0.32–1.59	0.32–3.27
PEF, L/min^a^				
*N*	17	50	50	117
Mean (SD)	325.0 (110.5)	269.6 (101.8)	145.5 (67.6)	224.6 (114.2)
Median	320.0	255.5	153.0	219.0
Minimum–maximum	3–478	89–492	50–287	3–492
Degree of breathlessness, *n* (%)^b^				
Not troubled except on strenuous exercise	0	0	0	0
Short of breath when hurrying on the level or walking up a slight hill	0	0	5 (10)	5 (3)
Walks slower than most people on the level, stops after a mile or so, or stops after 15 minutes walking at own pace	0	0	22 (44)	22 (15)
Stops for breath after walking about 100 yards or after a few minutes on level ground	0	0	19 (38)	19 (13)
Too breathless to leave the house, or breathless when undressing	0	0	4 (8)	4 (3)
Missing	50 (100)	50 (100)	0	100 (67)
GINA symptom control, *n* (%)^c^				
Well-controlled	14 (28)	1 (2)	0	15 (10)
Partly controlled	17 (34)	6 (12)	0	23 (15)
Uncontrolled	19 (38)	43 (86)	0	62 (41)

^a^ Spirometry tests were not performed by children <12 years of age (*n* = 33).

^b^ Participants with COPD completed the Medical Research Council Breathlessness Score.

^c^ Adults and children with asthma were assessed for symptom control according to GINA recommendations.

COPD, chronic obstructive pulmonary disease; FEV_1_, forced expiratory volume in 1 second; FVC, forced vital capacity; GINA, Global Initiative for Asthma; PEF, peak expiratory flow; SD, standard deviation.

### Primary inhalation parameters

Inhalation profiles were measured for 150 participants. The Digihaler data from one adult participant with asthma and one with COPD could not be downloaded. One child with asthma could only manage one inhalation and although another child made three valid inhalations only one could be used because their PIF measured by the IPR was below the 18 L/min minimum threshold for the Digihaler. Therefore, inhalation data were available for 441 inhalations, with paired data for 148 participants (50 children and 49 adults with asthma, and 49 adults with COPD). Of the 441 valid inhalations recorded, 12 had a PIF between 18 and 29 L/min using the Digihaler. Of the 148 paired data records, one child with asthma and two adults with COPD had PIF values between 18 and 29 L/min.

PIF data for each group and all participants are shown in Table 2. The mean percentage differences between Digihaler and IPR PIF measurements ranged from −2.97% among adults with COPD to 0.16% among children with asthma. Inhalation data categorized according to the PIF are presented in Supplementary Table S1.

**Table 2.** Inhalation Parameters Measured by the Digihaler and Inhalation Profile Recorder Using the Fastest Peak Inspiratory Flow from the Inhalation Profile

Parameter, mean (SD)	Children with asthma (*n* = 50)	Adults with asthma (*n* = 49)	Adults with COPD (*n* = 49)	All patients (*N* = 148)
PIF (Digihaler), L/min	65.63 (16.37)	82.12 (15.42)	64.20 (15.74)	70.62 (17.73)
PIF (IPR), L/min	66.67 (18.02)	84.85 (17.79)	66.27 (16.63)	72.55 (19.42)
Mean difference, L/min	−1.04	−2.72	−2.08	−1.94
95% CI	−3.32 to 1.24	−4.07 to −1.37	−2.84 to −1.31	−2.85 to −1.03
Mean difference, %	0.16	−2.47	−2.97	−1.75
95% CI	−5.05 to 5.36	−4.57 to −0.36	−3.99 to −1.95	−3.64 to 0.15
inhV (Digihaler), L	1.17 (0.54)	1.96 (0.70)	1.58 (0.58)	1.57 (0.69)
inhV (IPR), L	1.24 (0.54)	2.10 (0.77)	1.67 (0.59)	1.67 (0.73)
Mean difference, L	−0.07	−0.14	−0.09	−0.10
95% CI	−0.11 to −0.04	−0.21 to −0.07	−0.12 to −0.07	−0.13 to −0.07
Mean difference, %	−6.84	−6.03	−5.44	−6.11
95% CI	−12.14 to −1.54	−8.28 to −3.78	−7.15 to −3.73	−8.08 to −4.13
Ti (Digihaler), seconds	1.41 (0.57)	1.92 (0.60)	2.12 (0.76)	1.81 (0.71)
Ti (IPR), seconds	1.62 (0.74)	2.04 (0.73)	2.29 (0.84)	1.98 (0.82)
Mean difference, seconds	−0.21	−0.12	−0.17	−0.17
95% CI	−0.32 to −0.09	−0.23 to −0.02	−0.22 to −0.11	−0.22 to −0.11
Mean difference, %	−10.66	−4.31	−6.64	−7.23
95% CI	−15.49 to −5.84	−7.32 to −1.30	−8.91 to −4.36	−9.28 to −5.17
Tp (Digihaler), seconds	0.51 (0.34)	0.44 (0.30)	0.48 (0.26)	0.48 (0.30)
Tp (IPR), seconds	0.67 (0.39)	0.52 (0.36)	0.58 (0.33)	0.59 (0.36)
Mean difference, seconds	−0.16	−0.08	−0.10	−0.11
95% CI	−0.22 to −0.09	−0.14 to −0.02	−0.15 to −0.05	−0.15 to −0.08
Mean difference, %	−19.67	−7.20	−11.38	−12.80
95% CI	−27.25 to −12.09	−15.24 to 0.84	−18.83 to −3.93	−17.22 to −8.38

CI, confidence interval; inhV, inhalation volume; IPR, inhalation profile recorder; PIF, peak inspiratory flow; Ti, inhalation time; Tp, time to PIF.

**FIG. 4.**
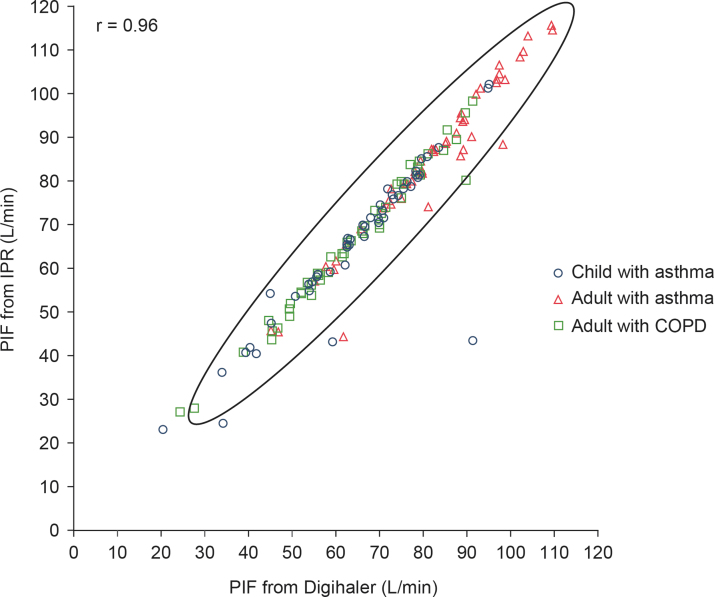
Scatter plot showing Spearman's correlation coefficient of highest PIF of each participant as measured by the Digihaler and its paired IPR value (*n* = 148 participants). Oval shown is 95% prediction ellipse, the region for predicting a new observation in the population and the approximated region that contains 95% of the population. COPD, chronic obstructive pulmonary disease; IPR, inhalation profile recorder; *r*, Spearman's correlation coefficient.

**FIG. 5.**
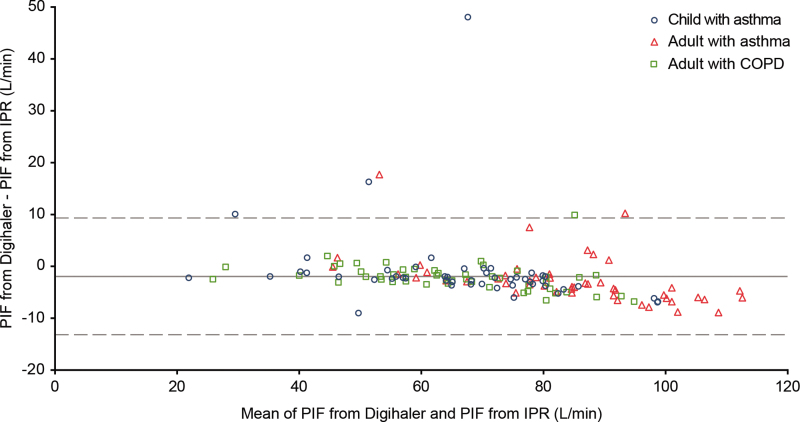
Bland–Altman scatter plot of the differences in highest PIF of each participant as measured by the Digihaler and its paired IPR value (*n* = 148 participants). Solid line represents the mean of differences (PIF from Digihaler minus PIF from IPR) and dotted lines represent ±2 SD of the differences. SD, standard deviation.

A scatter plot of the 148 paired PIFs is shown in Figure 4; the Spearman's correlation coefficient was 0.96 (95% CI: 0.94 to 0.97). Minimum and maximum Digihaler-measured PIF were 20.8 and 109.7 L/min, respectively. The Bland–Altman scatter plot of the difference in measurements is shown in Figure 5. A large difference between the PIF measured by the Digihaler and IPR for one participant was seen in a 4-year-old girl with asthma: PIF measured by the Digihaler was 91.5 L/min and by the IPR was 43.5 L/min. This suggests a partial air vent blockage by the upper lip during the inhalation. The participant's other inhalations showed only small differences between the Digihaler and IPR. Correlations and Bland–Altman plots for all 441 paired inhalations are presented in Supplementary Data.

Upon *post hoc* analysis, the mean Bayesian posterior percentage differences (95% credible intervals) between the Digihaler- and IPR-measured PIF among children with asthma, adults with asthma and adults with COPD were −3.88% (−4.45 to −3.29), −4.50% (−5.09 to −3.90), and −3.65% (−4.40 to −2.89), respectively.

### Secondary inhalation parameters

**FIG. 6.**
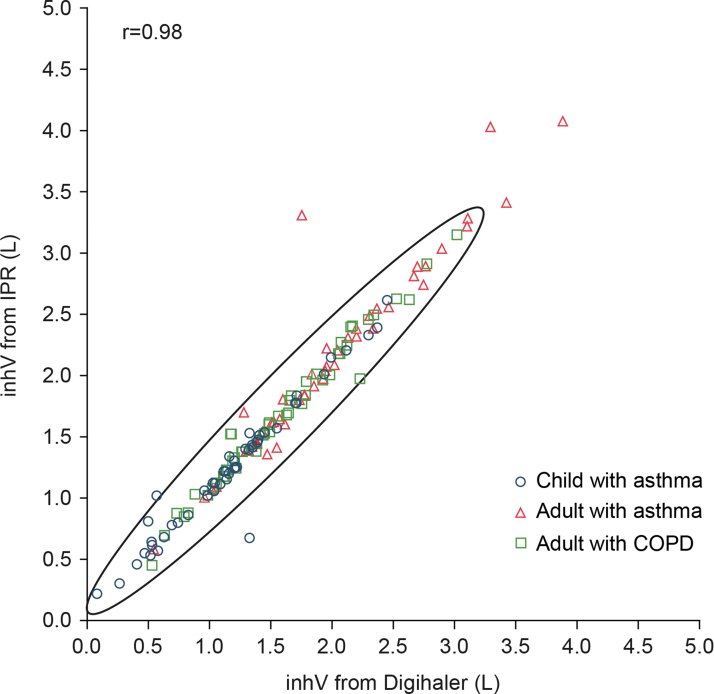
Scatter plot showing Spearman's correlation coefficient of inhV (from the profile with the highest PIF of a participant) as measured by the Digihaler and its paired IPR value (*n* = 148 participants). Oval shown is 95% prediction ellipse, the region for predicting a new observation in the population and the approximated region that contains 95% of the population.

**FIG. 7.**
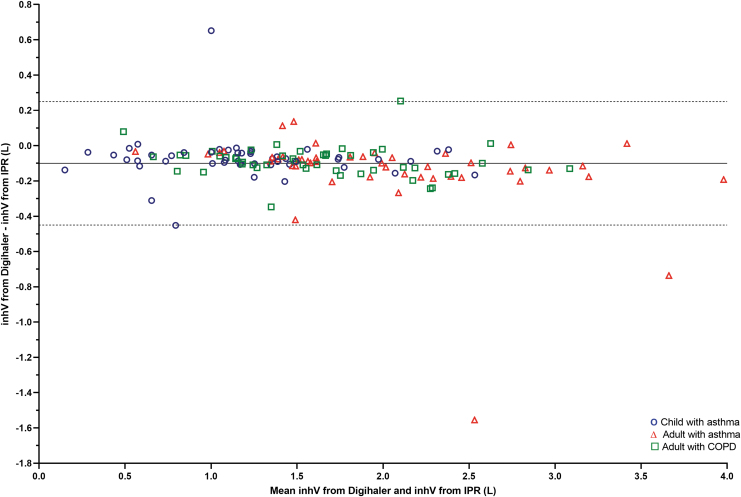
Bland–Altman scatter plot of the differences in inhV (from the profile with the highest PIF of a participant) as measured by the Digihaler and IPR (*n* = 148 participants). Solid line represents the mean of differences (inhV from Digihaler minus inhV from IPR) and dotted lines represent ±2 SD of the differences.

The mean percentage difference in inhV, measured by the Digihaler and IPR was −6.11 (95% CI: −8.08 to −4.13) (Table 2), ranging from −6.84% (95% CI: −12.14 to −1.54) among children with asthma to −5.44% (95% CI: −7.15 to −3.73) among adults with COPD (Table 2). The correlation between Digihaler- and IPR-measured inhV is shown in Figure 6. Mean inhV by PIF category are shown in Supplementary Table S1. Digihaler-measured inhV were slightly lower than with the IPR but were within −12.1% of the IPR for children with asthma, and −8.3% and −7.2% for adults with asthma and COPD, respectively. Bland–Altman analysis of inhV for the 148 pairs is presented in Figure 7. Correlations and Bland–Altman plots for all 441 inhalations are presented in Supplementary Data.

The overall mean percentage difference between the Digihaler and IPR measurements of Ti was −7.23% (95% CI: −9.28 to −5.17) (Table 2). Ti measurements by the Digihaler were lower than those recorded by the IPR but were within −15.5% of the IPR for children with asthma, and −7.3% and −8.9% for adults with asthma and COPD, respectively.

The mean percentage difference in Digihaler- and IPR-measured Tp was −12.80 (95% CI: −17.22 to −8.38) (Table 2). Mean percentage differences ranged from −19.67% (95% CI: −27.25 to −12.09) among children with asthma to −7.20% (95% CI: −15.24 to −0.84) among adults with asthma (Table 2). Digihaler-measured Tp were lower than those by the IPR but were within −27.3% of the IPR for children with asthma, and −15.2% and −18.8% for adults with asthma and COPD, respectively. Additional results can be found in Supplementary Table S2.

### Accuracy of the IPR to measure PIF

The mean (standard deviation) difference at nominal flows of 30, 60, and 90 L/min for IPR2 (*n* = 9 inhalers for each flow) were 1.28 (0.24), 2.57 (0.54), and 2.21 (0.77) L/min, respectively. These differences were 1.76 (0.73), 2.73 (0.39), and 2.43 (0.54) L/min for IPR3 (*n* = 10 inhalers for each flow).

## Discussion

There is a strong correlation between the PIF measurements by the Digihaler and those by the IPR; Digihaler-measured PIF was within 3% of PIF measured using the IPR. These findings confirm the potential of the Digihaler to provide reliable, objective data on inhalation technique and usage in a real-life setting.

There were four cases where discrepancies between Digihaler- and IPR-measured PIF were seen. This was possibly due to partial air vent blockage or incorrect pairings made by the programming. When a partial air vent block occurs, the pressure changes inside the eModule (measured by the Digihaler) are greater than those measured (by the IPR) at the mouthpiece. While deaggregation in DPIs is dependent on airflow through the device,^(21)^ the dose administered by the Digihaler will not be affected by a partial blockage as the pressure drop within the inhaler remains unaltered; therefore, partial air vent block was not regarded as a critical inhalation error. In addition, *post hoc* Bayesian analyses of the endpoints indicated that although outliers slightly increased the mean percentage differences in inhalation parameter measurements, this difference did not change the overall pattern of similarity between the Digihaler and IPR.

In general, higher inhalation flow rates are associated with improved DPI performance and a larger dose of inhaled medication.^(37,38)^ At lower inspiratory flow rates, the interaction between the inhalation flow and the inhaler's internal resistance to generate the turbulent airflow energy needed to deaggregate the dose is reduced.^(15)^ The inhalation threshold is unique to each DPI due to differing levels of resistance, internal designs, and powder formulation.^(39)^ For the Digihaler, a PIF threshold of 30 L/min has been suggested as the minimal requirement to ensure an adequate albuterol dose inhalation.^(40)^ In this study, almost all participants generated clinically sufficient PIF through the Digihaler that exceeded 30 L/min and most participants generated a PIF of ≥60 L/min following correct inhalation technique training. Only two inhalations (made by the same participant, a 7-year-old girl with asthma) had a PIF measured by the IPR of <18 L/min and these were recorded as zero by the Digihaler. This corresponds to an incidence of 0.45%. Therefore, during routine use in clinical practice, when a patient's PIF with the Digihaler records a zero flow, then this is most certainly due to the cap being opened, activating the eModule, and then closed without an inhalation being made.

Percentage differences between Digihaler- and IPR-measured inhV were small. Accurate determination of inhV is important since inhV has previously been shown to have significant effects on aerosol generation regarding mass output and particle size distribution in DPIs.^(41)^ Tp and Ti measurements with the Digihaler were numerically lower than those measured with the IPR. Underestimation of Tp could have resulted from flat inhalation profiles, that is, lacking a distinct PIF. For such profiles, the slightest increase in flow will be recorded as the PIF. Normal small variability of flow measurements by either the IPR or Digihaler can largely influence when the Tp occurs and therefore, Tp could have been detected at different points by the two devices. The underestimation of inhV and Ti, as measured by the Digihaler, may be explained by the low-flow threshold for the Digihaler (18 L/min). The IPR will have continued to detect an inhalation signal at flow rates down to 0 L/min among participants who kept inhaling at low flow rates. The Digihaler calculates inhV and Ti based on a truncated area under the curve between the start and end of the flow and so differences between the devices would be more noticeable at low flow rates.

Other digital inhalers that measure inhalation profiles are available or in development.^(15)^ The Inhaler Compliance Assessment (INCA) eModule, attached to the Diskus DPI, measures inhalation profiles using acoustic technology.^(42)^ The INCA device logs the date and time of use and stores a recording of the inhalation acoustics. This can then be downloaded but must be manually assessed in a laboratory. A previous study assessed inhalation profiles using acoustically determined PIF (PIFRc) measured by INCA and compared with accepted external methods.^(42)^ PIFRc measured with the eModule of INCA was correlated with spirometrically determined values (coefficient of determination = 0.88), with limits for the absolute difference of −11.9 to 19.4.^(42)^ As noted by Chrystyn et al., smaller mean differences between inhalation profiles measured with the Digihaler and those with accepted external methods were observed for the Digihaler compared with INCA.^(15)^ This suggests that measuring PIF using changes during an inhalation is more accurate using pressure sensors than acoustic methods.

In addition to their use in DPIs, audio-based methods have been used to objectively assess pressurized MDI (pMDI) patient technique to identify critical errors. Taylor et al. used the INCA device to record inhaler audio signals from 62 respiratory patients when using a pMDI.^(43)^ They found that 89% of patients made at least one critical technique error, even after tuition from an expert clinical reviewer. This demonstrates the value of inhaler monitoring systems to objectively monitor patient inhaler technique compared with standard checklist methods.

Digital solutions have a range of potential benefits. Previous studies have reported that patients who received feedback derived from data captured by digital inhaler systems demonstrated improved medication adherence.^(44–48)^ Furthermore, digital solutions could be used to help patients understand real-life inhaler usage.^(49,50)^ This may be useful in counseling sessions to discuss inhalation technique and self-management.^(15,25)^ Inhalation parameters recorded by digital inhalers could also alert physicians to changing patterns of dose usage that may warrant further discussion with patients. For example, it may be possible for digital inhalers to detect loss of disease control and predict exacerbations at an early stage,^(15,24,25)^ allowing for implementation of preventative plans.^(26)^ As these technologies develop, it will also be necessary to establish guidelines for their implementation and integration into existing health care systems.

This study had several limitations. First, calculation of mean PIF was limited by methods of data collection and analysis. For example, four instances of possible partial air vent blockages contributed to a larger difference in mean PIF. In addition, a small sample size at PIF <45 L/min may have contributed to inaccuracies in calculations. The limitations of the Digihaler may also have led to inaccuracies. For example, the Digihaler cannot detect flows <18 L/min, therefore leading to potential discrepancies between Digihaler and IPR measurements.

## Conclusion

In conclusion, findings from this study demonstrate a strong correlation between PIF and inhV measurements taken by the Digihaler and those taken by the IPR. Almost all study participants generated clinically sufficient PIF through the Digihaler to enable comparison and these data indicate that all participants, with the exception of possibly one, are able to generate flows adequate enough to ensure an acceptable albuterol dose. These findings, therefore, demonstrate that data provided by the Digihaler are accurate and clinically relevant, with the potential to assist patients and health care professionals in treatment decisions related to inhaler use and technique.

## Supplementary Material

Supplemental data

Supplemental data

Supplemental data

Supplemental data

Supplemental data

Supplemental data

Supplemental data

## Notes

Reviewed by:

Andy Clark

Chris O'Callaghan
