# Supplementary material for: Investigating the Accuracy of the Digihaler, a New Electronic Multidose Dry-Powder Inhaler, in Measuring Inhalation Parameters
Source: J Aerosol Med Pulm Drug Deliv. 2022 Jun 10;35(3):166–77. doi: 10.1089/jamp.2021.0031 (PMC9242715; doi:10.1089/jamp.2021.0031)
Supplement: Supplemental data [file Suppl_Data.docx]

**Supplementary materials**

***1.1 Safety***

Safety was assessed according to occurrence of adverse incidents, adverse events, and serious adverse events occurring during the time-period after the participant signed informed consent through to the end of study period (including the follow-up period).

There were no deaths, serious adverse events, adverse incidents, or withdrawals due to adverse events. Two children with asthma experienced one mild adverse event each (dizziness and asthma), unrelated to the study procedures. All vital signs fell within the normal range expected for the respiratory categories.

***1.2 Inhalation parameters***

Verbal training of the correct inhalation technique involved participants: 1) emptying their lungs as far as comfortable; 2) placing the inhaler in their mouth and sealing their lips, being careful not to block the vents; 3) inhaling as fast as they could for as long as they could; 4) taking the inhaler out of their mouth and holding their breath for as long as it felt comfortable.

## *1.3 Accuracy of the inhalation profile recorder to measure peak inspiratory flow*

Vacuum flow was provided to a critical flow unit (TPK 2000, Copley Scientific Ltd, Nottingham, UK) which was connected to a digital flow meter (DFM2000, Copley Scientific Ltd, Nottingham, UK) and a dosage unit sampling apparatus (DUSA) (Copley Scientific Ltd, Nottingham, UK). A disposable, clean, individually wrapped, single-participant use adapter was fitted onto the mouthpiece of the empty Digihaler and placed into the mouthpiece adapter of the DUSA. The side arm of the disposable mouthpiece adapter was connected to the inhalation profile recorder (IPR).

The critical flow unit was programmed to provide inhalations with nominal flows of 30, 60 and 90 L/min and an inhaled volume of 4 L through the Digihaler *in situ* in a dose unit sampling apparatus (Copley Scientific Ltd, Nottingham, UK). Actual flow and peak inspiratory flow (PIF) were measured by a digital flow meter (DFM2000, Copley Scientific Ltd, Nottingham, UK) and the IPR, respectively.

- 1. ***Statistical and analytical methods***

The relationships between PIF and peak expiratory flow, and between forced expiratory volume in 1 second and inhalation volume, were determined using the Spearman’s Rank Correlation Coefficient.
