# Supplementary material for: Investigating the Accuracy of the Digihaler, a New Electronic Multidose Dry-Powder Inhaler, in Measuring Inhalation Parameters
Source: J Aerosol Med Pulm Drug Deliv. 2022 Jun 10;35(3):166–77. doi: 10.1089/jamp.2021.0031 (PMC9242715; doi:10.1089/jamp.2021.0031)
Supplement: Supplemental data [file Suppl_TableS1.docx]

## Supplementary table 1. Inhalation parameters measured by the Digihaler and IPR in the ITT analysis set by PIF category

| Parameter, mean (SD) | PIF <30 L/min  (n=3) | PIF 30 to <45 L/min (n=6) | PIF 45 to <60 L/min (n=29) | PIF ≥60 L/min  (n=110) |
| --- | --- | --- | --- | --- |
| PIF (Digihaler), L/min  PIF (IPR), L/min  Mean difference, L/min  95% CI  Mean difference, %  95% CI | 24.43 (3.55)  26.04 (2.65)  -1.60  -4.85, 1.65  -6.40  -19.32, 6.52 | 38.37 (3.27)  37.39 (6.66)  0.98  -3.90, 5.87  5.08  -13.92, 24.08 | 52.46 (4.97)  53.96 (5.42)  -1.50  -2.29, -0.72  -2.65  -4.12, -1.18 | 78.42 (12.31)  80.64 (14.60)  -2.22  -3.42, -1.03  -1.75  -4.14, 0.64 |
| inhV (Digihaler), L | 0.91 (0.74) | 0.85 (0.52) | 1.26 (0.58) | 1.70 (0.67) |
| inhV (IPR), L | 1.11 (0.77) | 0.95 (0.57) | 1.34 (0.58) | 1.81 (0.72) |
| Mean difference, L | -0.20 | -0.10 | -0.08 | -0.11 |
| 95% CI | -0.52, 0.12 | -0.17, -0.03 | -0.11, -0.05 | -0.14, -0.07 |
| Mean difference, % | -30.89 | -10.92 | -6.66 | -5.02 |
| 95% CI | -102.78, 41.01 | -16.26, -5.57 | ‑9.90, -3.41 | ‑7.30, -2.75 |
| Ti (Digihaler), seconds | 2.53 (1.93) | 1.72 (1.06) | 1.92 (0.84) | 1.77 (0.60) |
| Ti (IPR), seconds | 2.93 (1.86) | 2.23 (1.68) | 2.14 (0.86) | 1.90 (0.69) |
| Mean difference, seconds | -0.40 | -0.51 | -0.22 | -0.13 |
| 95% CI | -1.17, 0.36 | -1.37, 0.35 | -0.32, -0.12 | -0.18, -0.07 |
| Mean difference, % | -26.52 | -15.54 | -10.04 | -5.50 |
| 95% CI | -105.83, 52.79 | -31.09, 0.02 | ‑14.85, -5.23 | ‑7.63, -3.38 |
| Tp (Digihaler), seconds | 0.52 (0.58) | 0.65 (0.50) | 0.55 (0.37) | 0.45 (0.25) |
| Tp (IPR), seconds | 0.67 (0.50) | 0.97 (0.56) | 0.75 (0.42) | 0.52 (0.31) |
| Mean difference, seconds | -0.14 | -0.33 | -0.19 | -0.08 |
| 95% CI | -1.05, 0.76 | -0.76, 0.11 | -0.29, -0.10 | -0.11, -0.05 |
| Mean difference, % | -6.39 | -32.98 | -23.16 | -9.14 |
| 95% CI | -213.57, 200.79 | -63.61, -2.35 | -31.66, -14.65 | -13.91, -4.37 |

CI, confidence interval; inhV, inhalation volume; IPR, inhalation profile recorder; ITT, intention-to-treat; PIF, peak inspiratory flow; SD, standard deviation; Ti, inhalation time; Tp, time to PIF.
