# Supplementary material for: Investigating the Accuracy of the Digihaler, a New Electronic Multidose Dry-Powder Inhaler, in Measuring Inhalation Parameters
Source: J Aerosol Med Pulm Drug Deliv. 2022 Jun 10;35(3):166–77. doi: 10.1089/jamp.2021.0031 (PMC9242715; doi:10.1089/jamp.2021.0031)
Supplement: Supplemental data [file Suppl_TableS2.docx]

## Supplementary table S2. Correlations between PIF and PEF, and between FEV_1_ and inhV, as measured by the Digihaler and IPR (ITT analysis set)

| Spearman’s Rank Correlation Coefficient | Children with asthma (n=17) | Adults with asthma (n=49) | Adults with COPD (n=49) | Total (N=115) |
| --- | --- | --- | --- | --- |
| **PIF and PEF** |  |  |  |  |
| From Digihaler | 0.79 | 0.30 | 0.13 | 0.40 |
| From IPR | 0.79 | 0.29 | 0.11 | 0.38 |
| **FEV_1_ and inhV** |  |  |  |  |
| From Digihaler | 0.70 | 0.44 | 0.45 | 0.37 |
| From IPR | 0.66 | 0.48 | 0.43 | 0.38 |

COPD, chronic obstructive pulmonary disease; FEV_1_, forced expiratory volume in 1 second; inhV, inhalation volume; IPR, inhalation profile recorder; ITT, intention-to-treat; PEF, peak expiratory flow; PIF, peak inspiratory flow.
