# Supplementary material for: Investigating the Accuracy of the Digihaler, a New Electronic Multidose Dry-Powder Inhaler, in Measuring Inhalation Parameters
Source: J Aerosol Med Pulm Drug Deliv. 2022 Jun 10;35(3):166–77. doi: 10.1089/jamp.2021.0031 (PMC9242715; doi:10.1089/jamp.2021.0031)
Supplement: Supplemental data [file Suppl_FigureS3.docx]

**Supplementary Figure S3. Scatter plot showing Spearman correlation coefficient of inhV for all inhalations as measured by the Digihaler and its paired IPR value (n=441 inhalations)**


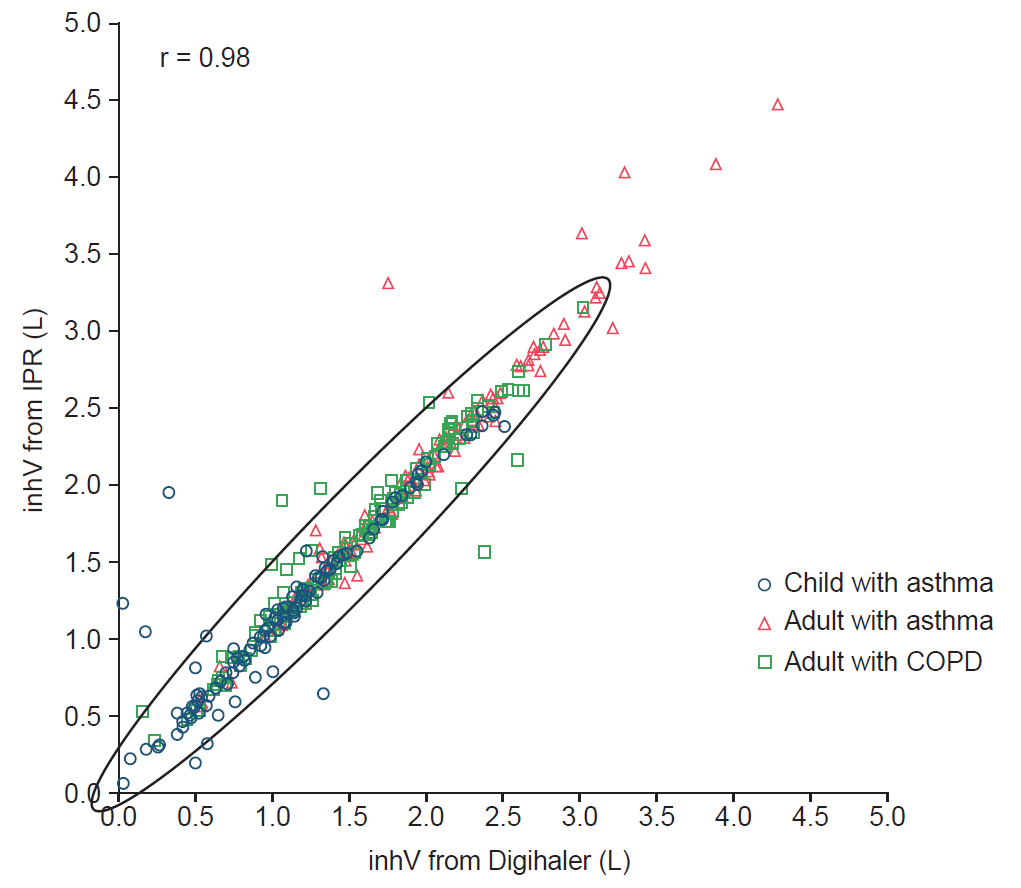


Oval shown is 95% prediction ellipse, the region for predicting a new observation in the population and the approximated region that contains 95% of the population.

COPD, chronic obstructive pulmonary disease; inhV, inhalation volume; IPR, inhalation profile recorder; r, Spearman correlation coefficient.
