# Supplementary material for: Investigating the Accuracy of the Digihaler, a New Electronic Multidose Dry-Powder Inhaler, in Measuring Inhalation Parameters
Source: J Aerosol Med Pulm Drug Deliv. 2022 Jun 10;35(3):166–77. doi: 10.1089/jamp.2021.0031 (PMC9242715; doi:10.1089/jamp.2021.0031)
Supplement: Supplemental data [file Suppl_FigureS4.docx]

**Supplementary Figure S4. Bland-Altman scatter plot of the differences in inhV as measured by the Digihaler and its paired IPR value for all inhalations (n=441 inhalations)**


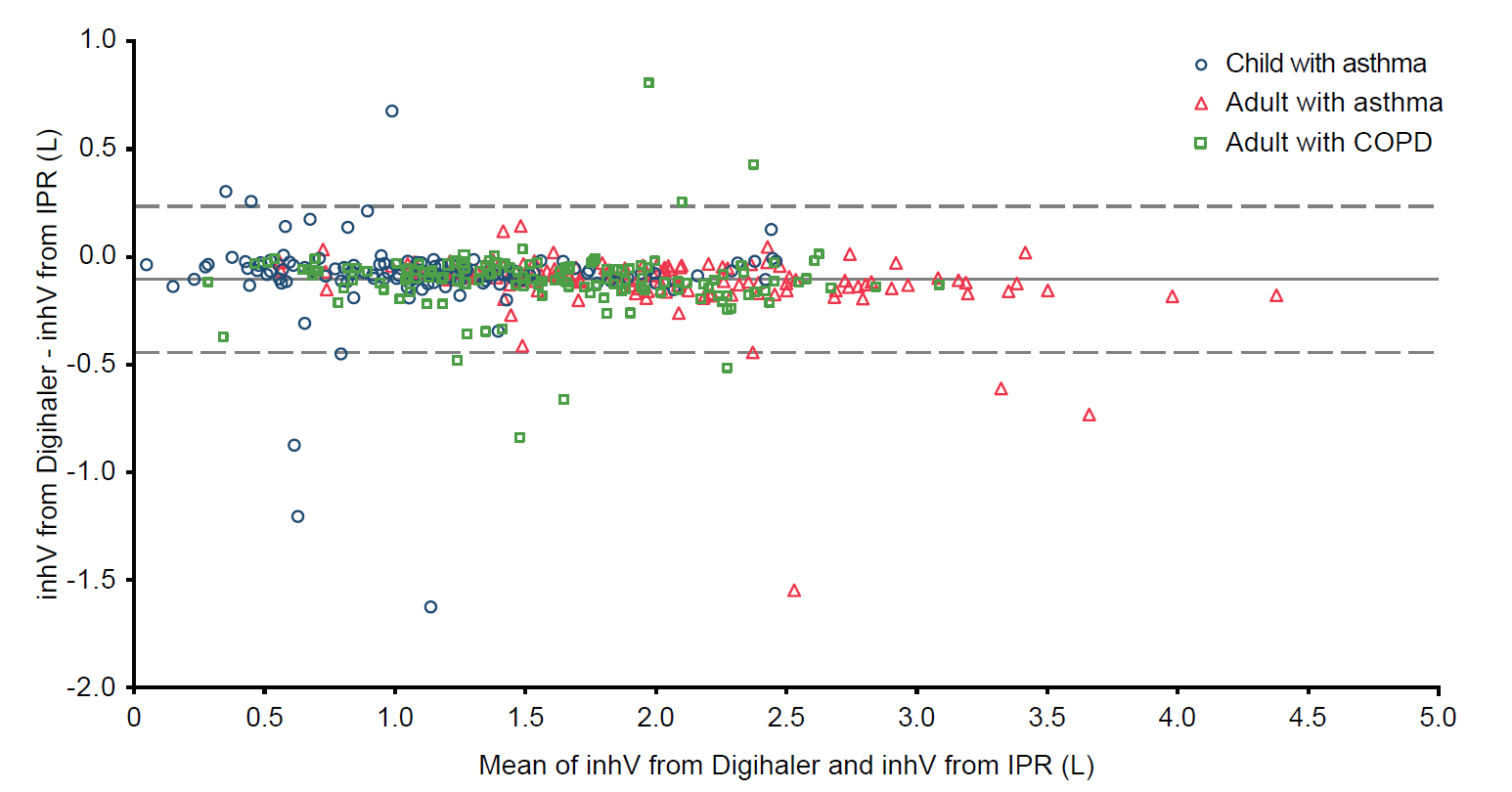


Solid line represents the mean of differences (inhV from Digihaler minus inhV from IPR) and dotted lines represent ±2 SD of the differences. Mean difference (95% CI): -0.10 (-0.12, -0.09) L.

CI, confidence interval; COPD, chronic obstructive pulmonary disease; inhV, inhalation volume; IPR, inhalation profile recorder; SD, standard deviation.
